# Pretreatment methods affecting the color, flavor, bioactive compounds, and antioxidant activity of jujube wine

**DOI:** 10.1002/fsn3.1793

**Published:** 2020-07-27

**Authors:** Wenchao Cai, Fengxian Tang, Chunhui Shan, Qiangchuan Hou, Zhendong Zhang, Yun Dong, Zhuang Guo

**Affiliations:** ^1^ School of Food Science Shihezi University Shihezi China; ^2^ Northwest Hubei Research Institute of Traditional Fermented Food School of Chemical Engineering and Food Science Hubei University of Arts and Sciences Xiangyang China

**Keywords:** antioxidant activity, bioactive compounds, color, electronic senses, Jujube wine, multivariate statistical analysis

## Abstract

In the case of wine production, the selection of optimal pretreatment methods and starter cultures are the 2 key points before fermentation. In this research, the fresh jujube was separately underwent alcoholic fermentation at 20°C with 3 different pretreatment methods (with peel, without peel, and juice) and 5 different starter cultures, respectively. Color analysis, electronic sense analysis, bioactive compound analysis, and antioxidant activity analysis combined with multivariate statistical analysis were applied to evaluated the effects of pretreatment methods and starter cultures on the overall quality of jujube wine. It was found that both pretreatment methods and starter cultures have effects on the quality of jujube wines, in which pretreatment methods have much more significant effects. The jujube wines fermented with different pretreatment methods were classified clearly by their overall quality, and that of the jujube wines fermented with peel was the best among all, since it can not only enhance the color and flavor quality of the wine, but also maximize the preservation of bioactive compounds and antioxidant activity of jujube for better consumer acceptance. This will provide a theoretical reference and application basis for the quality improvement of jujube wine.

## Graphical abstract

In the case of the wine production, the selection of optimal pretreatment methods and starter cultures are the 2 key points before fermentation. Hence, in this research, the effects of pretreatment methods and starter cultures on the quality of jujube wine were evaluated by multivariate statistical analysis methods from the aspects of color, aroma, flavor, bioactive compounds, and antioxidant activity. What’s more, the most appropriate pretreatment method for fresh jujube wine‐making was also identified, which provided a theoretical reference and application basis for the quality improvement of jujube wine.

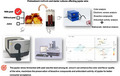

## 1 INTRODUCTION

Jujube (*Zizyphus jujuba* Miller) is a kind of fruit integrated with medicine and food that native to China with a history of more than 4,000 years (Li, Fan, Ding, & Ding, 2007). It is rich in sugar, fiber, minerals, proteins, organic acids, phenolic acids, carotenoids, vitamins (especially vitamin C), flavonoids, and cerebrosides with the medicinal value of preventing as well as treating tumors, cardiovascular diseases, anorexia, and fatigue (Hernandez et al., 2016). Thanks to its rich nutrition, unique flavor, and high utilization value, jujube is increasingly favored by consumers. However, the fresh jujube has problems such as difficulty in preservation, perishability, deterioration, and easy loss of nutrients after harvesting, which cause greater sales pressure to fruit farmers. Especially in recent years, with the production area and output of jujube expanding year by year, the fresh fruit market is gradually saturated and the production benefit is reduced. Therefore, the development of deep‐processing products such as jujube wine (JW) (Zhang, Zhang, & Xu, 2016), jujube brandy (Li et al., 2016), and jujube fermented juice (Cai et al., 2019; Cai et al., 2018) can not only alleviate the market pressure of fresh fruit sales, but also significantly increase the added value of products, improve farmers’ income, and promote the healthy and benign development of jujube industry.

Jujube is an excellent source of sugar and nutrient, which is very suitable for wine making (Lee, Yun, Lee, & Kim, 2018). JW fermented by yeasts is clear and yellowish, with strong fruity and alcoholic flavor, which is well loved by consumers (Lee et al., 2018). At present, JW is mainly fermented by juice, with low utilization rate and high cost (Li et al., 2019). Fermentation by peel and flesh is the basic technology of red wine making, which can transfer the polyphenols and flavor compounds from peel and flesh to wine as much as possible (Katalinic, Milos, Modun, Music, & Boban, 2004). Thus, fermentation with jujube peel and flesh can also transfer nutrients and flavor compounds from the raw material into products. Park, Suwanmanon, Towantakavanit, and Gorinstein (2011) have found that adding peel to fermentation broth can improve the aroma and antioxidant abilities of wine, and accelerate the dissolution of phenols and phenolic compounds, along with changing the appearance of wine and affecting the quality of wine (Amos, 2007; Perez‐Magarino & Jose, 2004). Long‐term consumption can prevent arteriosclerosis and coronary heart disease (Selli, Cabaroglu, Canbas, Erten, & Nurgel, 2003).

The quality of wine is related to many factors, from raw material selection, pretreatment methods, starter cultures to fermentation and storage. The aroma and taste are important perceptual representations for evaluating the wine quality, which are related to the sensory characteristics perceived by consumers and directly affect the overall evaluation of products by consumers (Vidal et al., 2020; Xu et al., 2016). Despite the fact that sensory evaluation is the most intuitive and rapid method to identify the aroma and taste of wine, it still has a high demand for evaluation personnel and inadequate evaluation index system (Marsanasco, Marquez, Wagner, Chiaramoni, & Alonso, 2015). Electronic nose (E‐nose) and electronic tongue (E‐tongue) are new food quality testing methods that mimic human olfactory and taste systems. They are widely used due to their portability, low price, good repeatability, and adaptability. However, human taste and smell are not independent and interact with each other. Therefore, E‐nose and E‐tongue techniques can be combined to detect food quality, which have achieved good results in some food fields. Di Natale et al. (2000) researched milk with different freshness by combined technique of E‐nose, E‐tongue, and principal component analysis (PCA). It turned out that the detection capacity after modeling with E‐technology response values was better than using raw data directly. Banerjee et al. (2012) tested black tea quality using E‐nose and E‐tongue in an individual or combined way, respectively. The results indicated that the combined one was better in performance. Haddi et al. (2014) used E‐nose and E‐tongue individually, and E‐nose and E‐tongue with PCA comprehensively to detect differently branded fruit and vegetable juices. The results suggested that the detection ability of joint method is better than using either technique alone. Therefore, it is of positive significance to introduce the above detection techniques into JW quality evaluation.

For consumers, in addition to the flavor of JW, antioxidant activity also represents the major parameters in determining the quality of JW. Oxidation is essential to many organisms, since it can generate energy and fuel biological processes (Zhang, Jiang, Ye, Ye, & Ren, 2010). However, the uncontrolled production of oxygen‐derived free radicals is hostile and harmful to cells along with their functions, and thus plays an important role in pathogenesis of cancer, cirrhosis, cardiovascular diseases, atherosclerosis, and inflammation and other diseases (Aruoma, 1998). In recent years, natural antioxidants with high contents of bioactive compounds such as fruits and vegetables have attracted wide attention because they can ameliorate oxidative damage induced by free radicals and are safer than synthetic antioxidants (Anagnostopoulou et al., 2006). Since jujube is a high‐quality natural antioxidant, the interest in evaluating its bioactive compounds and antioxidant activity has substantially increased and numerous studies have been undertaken. Xue, Feng, Cao, Cao, and Jiang (2009) measured the antioxidant activity of 3 jujube cultivars and revealed that the high antioxidant activity of jujube could be attributed to the high total phenolic content in the fruit. Gao et al. (2011) determined the bioactive compounds and antioxidant activity of 5 jujube cultivars. The results demonstrated that the cultivar is the main factor which influences the bioactive compounds and antioxidant activity of jujube. Han, Lee, Park, Ahn, and Lee (2015) optimized extraction conditions for jujube pulp and seed in order to obtain maximum bioactive compounds and antioxidant activity. Although there are extensive researches on jujube, nevertheless the bioactive compounds and antioxidant activity of JW are rarely reported. For this reason, as a deep‐processed product of jujube, it is necessary to evaluate the bioactive compounds contents and antioxidant activity of JW.

The aim of this research was to identify how pretreatment methods and starter cultures affecting the quality of JW as well as to determine the optimal process. Color, aroma, taste, bioactive compounds, and antioxidant activity of JW processed with different pretreatment methods and starter cultures were investigated with E‐nose, E‐tongue, and other instruments in this research. This will provide a reference for the subsequent processing, quality control, and marketing of JW.

## 2 MATERIALS AND METHODS

### 2.1 Raw material

The fresh and undamaged jujube (*Zizyphus jujuba* cv. Dongzao) was collected (October 2019) from a local market in Aksu, the Xinjiang Uygur Autonomous Region, China.

A total of 5 starter cultures (*Saccharomyces cerevisiae*) compromising AU, EC, and MA from Yantai DiBoshi brewing machine Co., Ltd., along with BV and RW from Angel Yeast Co., Ltd., were purchased (October 2019) online.

### 2.2 Preparation

The fresh jujube was cleaned by washing thoroughly in tap water twice, stoned, and processed with the following 3 different pretreatment methods (with peel, without peel, and juice), respectively, to obtain fermentation broth:

1. With peel (WP): The whole fresh jujube was blended in a high‐speed blender (L18‐Y928, Joyoung Co., Ltd.) for 30 s and then subjected to pectinase treatment (Lallemand Group Co., Ltd., 0.3 g/L, activated: 10,000 U/g) at 45°C for 2 hr.
2. Without peel (WOP): The whole fresh jujube was peeled and blended in a high‐speed blender for 30 s and then subjected to pectinase treatment at 45°C for 2 hr.
3. Juice (J): the jujube pulp obtained according to abovementioned pretreatment method WP was filtrated through an eight‐layer gauze to get jujube juice.

### 2.3 Fermentation

Sulfur dioxide (50 mg/L) was added into the fermentation broth followed by the separate inoculation with 0.03% (w/w) of 5 different starter cultures (AU, EC, MA, BV, and RW). The 15 fermentation broths prepared by combining 3 different treatment methods with 5 different yeast strains were fermented at 20 ± 1°C statically. During the fermentation, the alcohol content was monitored. When the alcohol content of the samples reached to the optimum alcohol content of 10% obtained from the previous experiment, the fermentation broth was filtrated through an eight‐layer gauze and centrifuged at 4°C, 6,000 *g* for 15 min to terminate the alcoholic fermentation and eliminate fruit particles from the jujube. The supernatant was bottled; then, the resulting JW was added with 0.4% bentonite and kept for another 3 days at room temperature for clarification prior to follow‐up analysis.

### 2.4 Color analysis

The color attributes including L* (lightness), a* (red/green), b* (yellow/blue), and luminousness were measured using the Ultrascan PRO HunterLab colorimeter (HunterLab) in the total transmission mode and UVmini‐1240 spectrophotometer (Shimadzu). The colorimeter was calibrated by a white standard tile (L* = 99.20, a* = −0.08, b* = −0.01), after which the samples were filled into cuvettes and the color attributes were recorded.

### 2.5 Electronic senses analysis

E‐nose analysis was performed with a Portable Electronic Nose (PEN3, Win Muster Airsense Analytics Inc.), consisting of 10 metal–oxide–semiconductor (MOS) type chemical sensors: W1C (aromatic compounds), W5S (broad‐range compounds, polar compounds, nitrogen oxides, and ozone), W3C (ammonia, aromatic compounds, aldehydes, ketones), W6S (hydrogen, broad‐range compounds), W5C (arom‐aliph, alkanes, aromatic compounds, less polar compounds), W1S (methane, broad‐methane, broad‐range compounds), W1W (sulfur compounds, terpenes, and sulfur organic compounds), W2S (alcohols, partially aromatic compounds, ketones), W2W (aromatic compounds, sulfur organic compounds), and W3S (methane‐aliph) (Cai et al., 2019). The sensor response is expressed as resistivity (Ohm).

E‐tongue analysis was applied with a commercial E‐tongue (Taste‐Sensing System SA 402B, Intelligent Sensor Technology Co. Ltd.), comprising 5 chemical sensors, who have different response properties to chemicals based on different tastes, namely CA0 specific for sourness, C00 for bitterness and aftertaste bitterness (aftertaste‐b), AE1 for astringency and aftertaste astringency (aftertaste‐a), CT0 for saltiness, and AAE for umami and richness.

Both E‐nose and E‐tongue analysis were conducted following a method described by Cai et al., (2020).

### 2.6 Bioactive compounds and antioxidant activity analysis

Total phenolic content (TPC) of JW was determined by applying a modified Folin–Ciocalteu method described by Najafabadi, Sahari, Barzegar, and Esfahani (2017) with minor modifications. Samples of 1 ml were mixed with 2 ml of Folin–Ciocalteu reagent. The mixture was vortexed for 1 min and then mixed with 2 ml of 7.5% (m/v) sodium carbonate solution. After being kept in the dark for 1 hr at room temperature, the absorbance of the resultant mixture was measured at 765 nm against blank on a UVmini‐1240 spectrophotometer. The results of TPC in the samples were reported as mg of gallic acid equivalent (GAE)/ml from a calibration curve constructed using standard solution of gallic acid.

Total flavonoid content (TFC) of JW was determined following a method by Gao et al. (2012) with slight modifications. In sum, in a 10‐ml test tube, 0.5 ml of extracts, 3.5 ml of 30% methanol, 0.4 ml of NaNO2 solution (5%, wt/vol), and 0.4 ml of Al(NO3)3 solution (10%, m/v) were mixed. After 6 min, 0.4 ml of NaOH (1 M) was added. The resultant solution was mixed well at room temperature for 15 min, and the absorbance was measured at 516 nm against blank. The total flavonoid content in the samples were expressed as mg of rutin equivalents (RE)/ml from a calibration curve constructed using the methanolic solution of standard rutin (0–100 mg/L) with the same procedure as earlier mentioned.

Total anthocyanin content (TAC) was determined by the pH differential method described by Najafabadi et al. (2017) with minor modifications. Specifically, 2 buffer solutions, KCl (0.025 M) at pH 1 and CH3COONa (0.4 M) at pH 4.5, were prepared. Then, 100 µl of each sample was distributed into 2 sets of tubes, and a 0.9 ml of the KCl buffer was distributed into 1 set of the tubes; meanwhile, 0.9 ml of CH3COONa was added to the other. The tubes were then vortexed and absorbance read at 520 nm and 700 nm, respectively, on UVmini‐1240 spectrophotometer against a blank. The TAC was calculated using equation 1 and expressed as mg of cyanidin 3‐glucoside equivalents (CE)/ml.

The 1,1‐diphenyl‐2‐picrylhydrazyl (DPPH) radical scavenging activity of JW was assayed followed the procedure of Gao et al. (2012) with slight modifications. In brief, each 0.02 ml sample was mixed with a freshly prepared solution of DPPH (1 mg, 2.4 mM in methanol). The mixture was vigorously shaken for 15 s and then kept at 37°C in the dark for 45 min. The absorbance was read against a blank at 519 nm on a UVmini‐1240 spectrophotometer. A calibration curve was performed using Trolox, and the antioxidant activity was reported as µmol of Trolox equivalents (TE)/L.

The 2, 2'‐azino‐bis (3‐ethylbenzothiazoline‐6‐sulfonic acid) (ABTS) radical cation scavenging activity was assayed using a published ABTS method by Gao et al. (2012) adopted with suitable modifications. The ABTS radical cation (ABTS•+) were formed by the reaction of 7.4 mM ABTS solution with 2.6 mM K2S2O8. The mixture was kept at room temperature in the dark for 12–16 hr. The ABTS•+ solution was diluted with ethanol to reach an absorbance of 0.70 ± 0.02 at 734 nm. The ABTS solution (100 µl), distilled water (80 µl), and sample (20 µl) were mixed, and then, the absorbance was recorded at 734 nm after 6 min. Trolox standard solution was used to perform the calibration curve, and the results were expressed as µmol of Trolox equivalents (TE)/L.

### 2.7 Statistical analysis

Samples were twice analyzed in triplicate experiments, and results were expressed as the mean value ± standard deviation (*SD*) prior to all calculations. Variance and significant difference tests were statistically analyzed by one‐way analysis of variance (ANOVA) and Duncan's multiple range tests. Significant difference was calculated at the 0.05 level. The analysis of fusion data was conducted multivariate statistical analysis methods (cluster analysis, principal coordinate analysis, principal component analysis, multivariate analysis of variance, and linear discriminant analysis).

Cluster analysis (CA), principal coordinate analysis (PCoA), principal component analysis (PCA), and linear discriminant analysis (LDA) were conducted with R software (version 3.6.1), while canonical correlation analysis (CCA) and multivariate analysis of variance (MANOVA) were performed by using the Data Processing System (DPS) (version 9.50, Hangzhou Ruifeng Information Technology Co., Ltd.) (Tang & Zhang, 2013). The figures were plotted by Origin (version 2019, Origin Lab) and R software.

## 3 RESULTS AND CONCLUSIONS

### 3.1 Diversity analysis

The perceptions in the choice and acceptance of a food product by consumers are determined by the appearance, aroma, and taste of the food (Judacewski et al., 2019). Based on the color analysis, electronic senses analysis, bioactive compound analysis, and antioxidant activity analysis of 15 JW samples (3 different pretreatment methods × 5 different starter cultures), a data matrix consisting of 15 rows (15 samples) and 27 columns (27 quality indexes compromising 4 color attributes, 10 aroma attributes, 8 taste attributes, 3 bioactive compounds attributes, and 2 antioxidant activity attributes) was obtained, after which multivariate statistical analysis was applied to evaluate the effects of pretreatment methods and starter cultures on JW.

**Figure 1:**
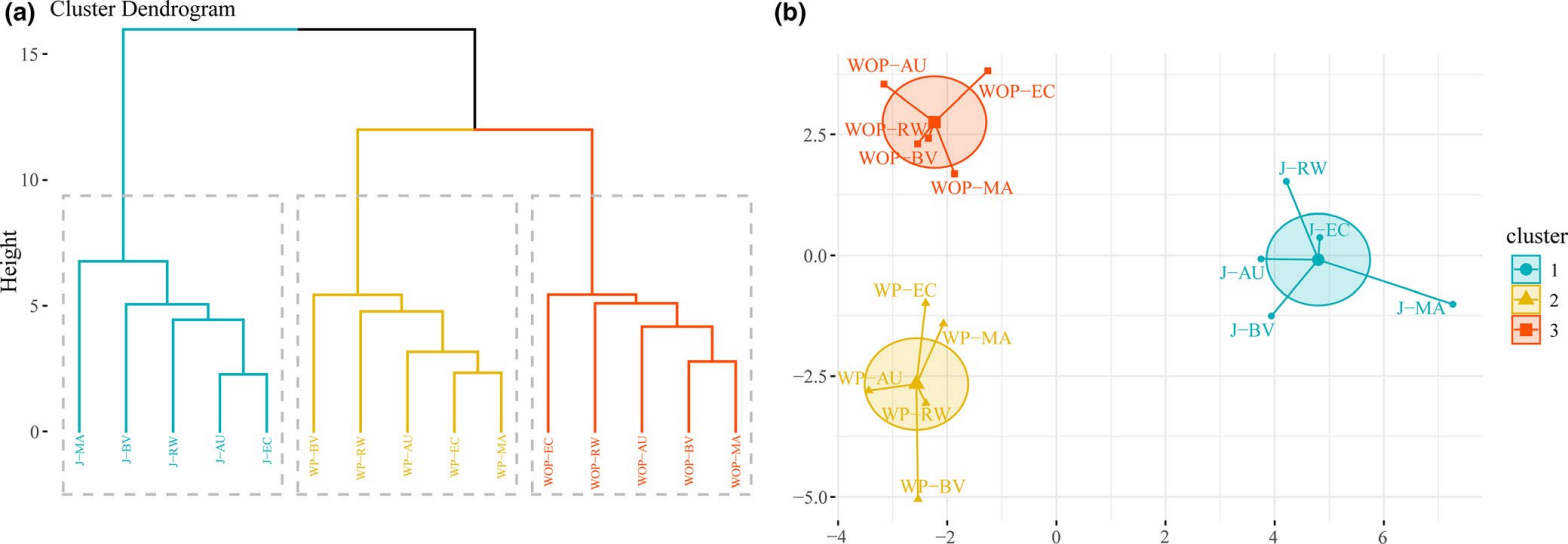
CA (a) and PCoA (b) plots of JW fermented by different pretreatment methods and different starter cultures

The JW samples were classified according to their overall quality by CA, an unsupervised explorative analysis based on unweighted pair group method with arithmetic mean (UPGMA) (Granato et al., 2018). It can be seen in Figure 1a that JW samples can be divided into 3 clusters as a whole. At the mean distance of 10, from right to left, cluster 1 contains all JW fermented WOP; cluster 2 contains all JW fermented WP; and cluster 3 contains all JW fermented by J.

Then, the sample clustering was visualized by PCoA in Figure 1b, which further confirmed that the samples with different pretreatment methods and different starter cultures were divided into 3 clusters. Thereinto, cluster 1 comprises all JW fermented WOP, cluster 2 comprises all JW fermented WP, and cluster 3 comprises all JW fermented by J, which is in consonance with the observations of CA based on UPGMA. It is also shown in Figure 1b that samples with the same starter culture fall into different clusters, it follows that starter culture is not the crucial factor to quality discrepancy of JW. In contrast, pretreatment methods may have greater impact on JW quality. Besides, JW fermented WP are situated relatively closer to JW fermented WOP instead of JW fermented by J, indicating that there are significant differences among the JW fermented with different pretreatment methods, and the overall quality of JW fermented WP are relatively similar to JW fermented WOP rather than JW fermented by J.

**Figure 2:**
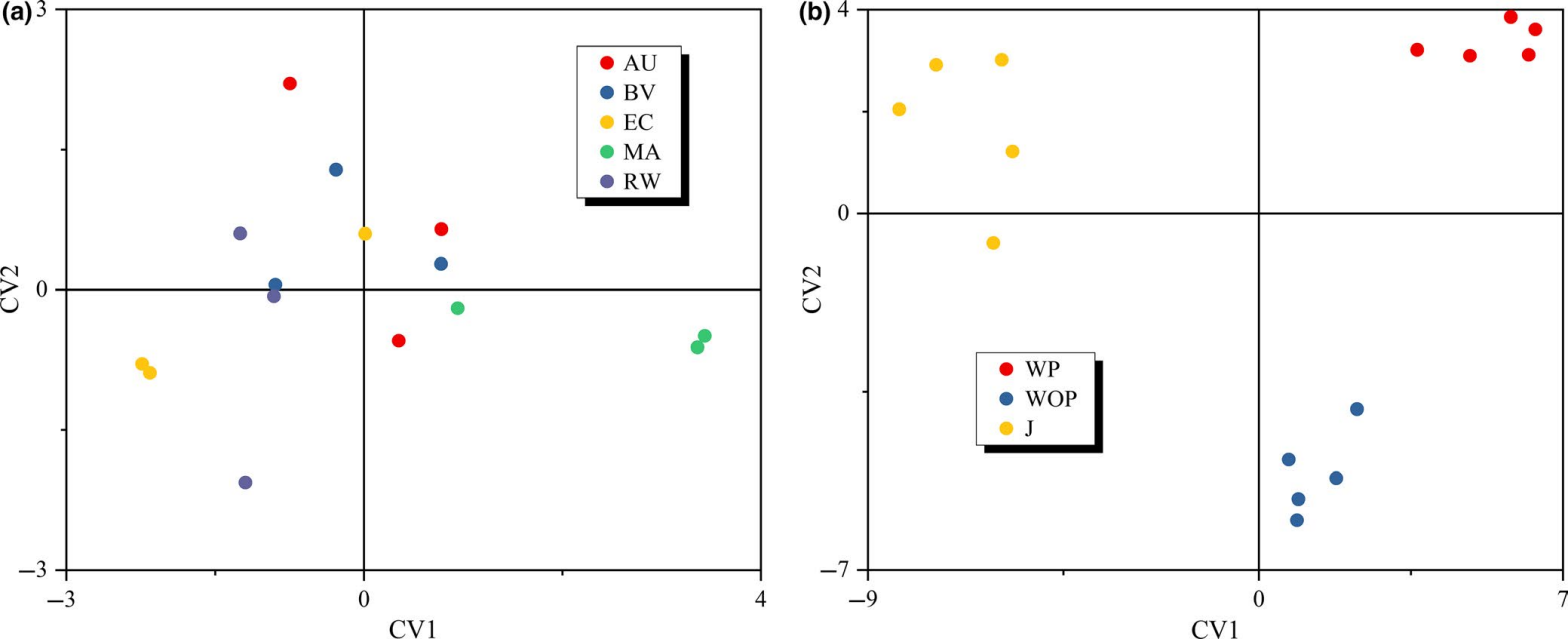
CCA similarity plots of JW fermented with different pretreatment methods (a) and different starter cultures (b) defined by canonical variates 1 and 2

To validate the qualitative results above and determine the influence of different pretreatment methods and different starter cultures on JW quality, a constrained statistical analysis method, CCA, was used based on the grouping information of JW fermented with different starter cultures, and the results are shown in Figure 2a. The spatial arrangement of all JW samples is continuous, and the dispersion trend is not obvious. Most samples are distributed irregularly and randomly. However, there are still some samples with weak clustering trend. For example, the JW fermented by MA are mainly distributed in the fourth quadrant. Meanwhile, considering the grouping information of JW fermented with different pretreatment methods, the spatial distribution of all samples was arranged, and the results are shown in Figure 2b. The JW fermented by different pretreatment methods showed obvious separation and clustering trends, indicating that their overall quality was quite different. The JW fermented WP are mainly distributed in the first quadrant, and the JW fermented by J are mainly distributed in the second along with third quadrant, while the JW fermented WOP are distributed in the fourth quadrant. Hence, it is qualitatively considered that different pretreatment methods have a notable impact on the overall quality of JW. And different starter cultures may also affect the JW quality; however, their impact is far less than that of different pretreatment methods. This illustrated that controlling and improving the JW quality would first require determination of optimal pretreatment method. Consequently, effects of pretreatment methods on JW quality were mostly investigated in the follow‐up analyses.

### 3.2 Color analysis

**Figure 3:**
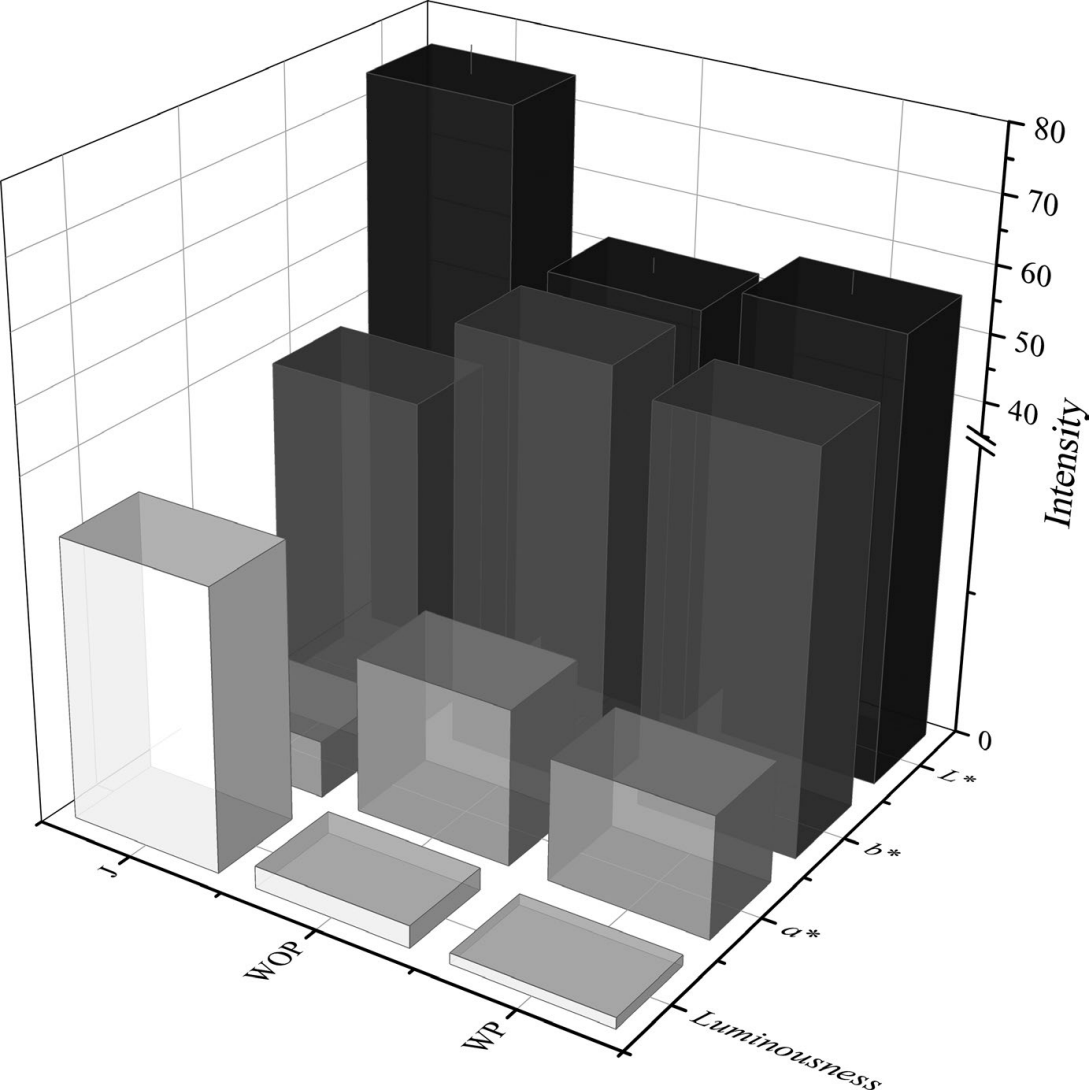
Color attributes of JW fermented by different pretreatment methods

The color attributes of JW fermented by different pretreatment methods are presented in Figure 3. The color attributes differ significantly (*p* < .05) among the JW samples. JW fermented by J possessed significant higher values (*p* < .05) on L* and luminousness, as well as significant lower values (*p* < .05) on a* and b*, compared to JW fermented by other pretreatment methods. As for JW fermented WP and JW fermented WOP, no significant (*p* > .05) difference in color between them was observed. This indicates that JW fermented by J was brighter with a more green and blue color, while JW fermented WP and JW fermented WOP were darker, more pigmented with a more red and yellow color. The red and yellow colors of yellow‐fleshed JW are important parameters for consumer acceptance; hence, JW fermented WP and JW fermented WOP possessed better color quality. This might be attributed by the involvement of pulp or peel during fermentation, which directly determines the leaching behaviors of pigment compounds in fresh jujube during fermentation and trigger the improvement in color quality. Similar trends have also been reported by Rommel, Wrolstad, and Heatherbell (1992), who revealed the color quality improvement capacity of pulp contact to wine. Additionally, many researches have shown that the color of wine mainly depends on TAC in the fruit and a* is significantly (*p* < .05) positively correlated with TPC (Ouyang et al., 2018; LAGOVANZELA et al., 2014; Wang et al., 2015). According to the previous research, total anthocyanins and total phenols have been identified as major natural antioxidants with many beneficial physicochemical and biological properties (Espada‐Bellido et al., 2017; Mendez‐Lagunas, Rodriguez‐Ramirez, Cruz‐Gracida, Sandoval‐Torres, & Barriada‐Bernal, 2017), thus suggesting a better potential antioxidant activity and health beneficial function of JW fermented WP and JW fermented WOP.

### 3.3 Electronic senses analysis

The mean response values of E‐nose and E‐tongue analysis were graphically displayed by means of a rose diagram.

**Figure 4:**
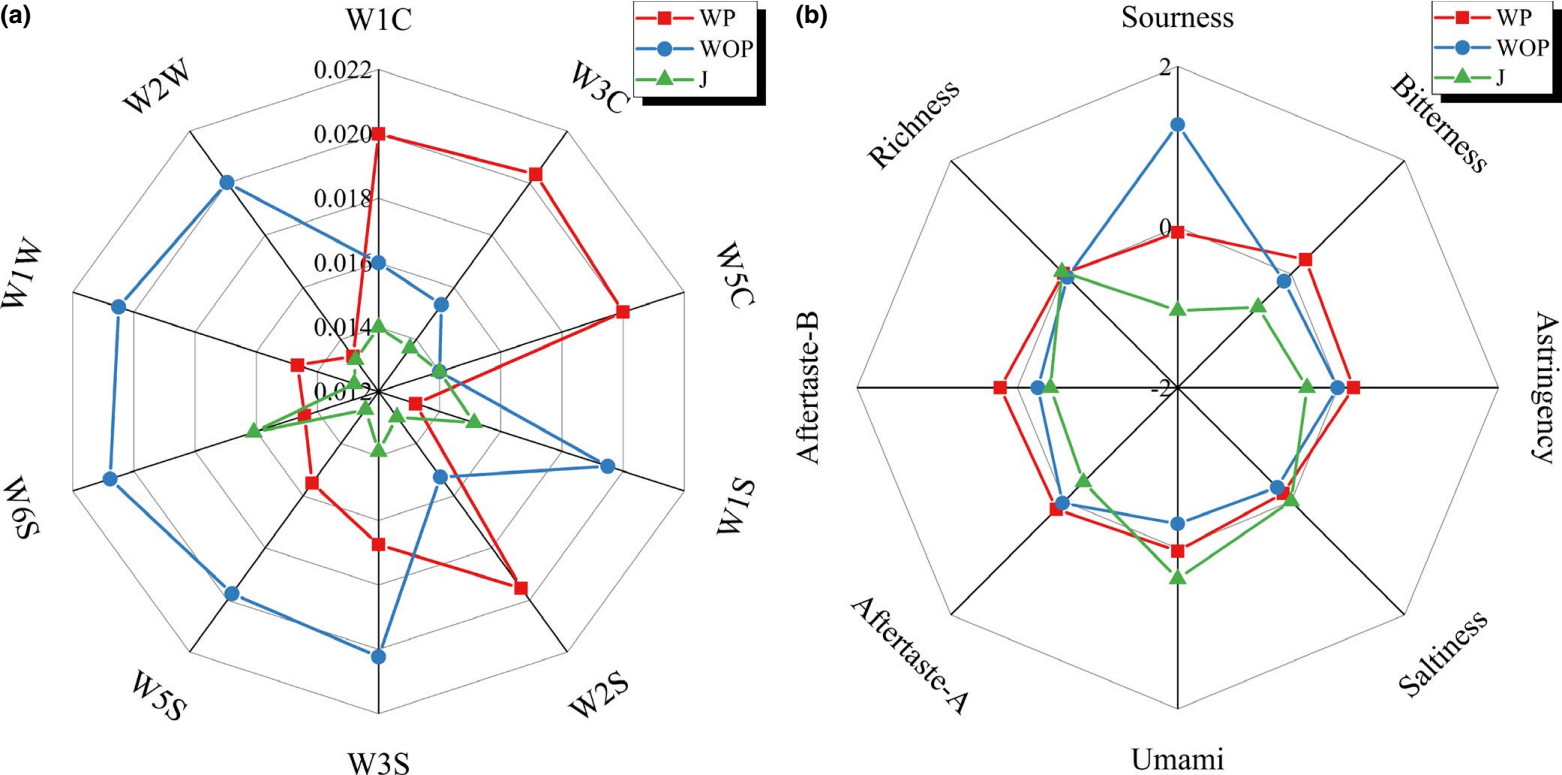
Rose diagram for E‐nose data of aroma (a) and E‐tongue data of taste (b) among JW fermented by different pretreatment methods

The E‐nose sensors are nonspecific, or semispecific for classes of compounds and the response value them is related to the chemical composition of volatile compounds; therefore, the E‐nose sensors were grouped into three categories: WC (W1C, W3C, and W5C) for aromatic compounds; WW (W1W and W2W) for sulfur organic compounds; and WS (W1S, W2S, W3S, W5S, and W6S) of broad‐range sensitivity (Buratti, Benedetti, & Giovanelli, 2017). As shown in Figure 4a, the response values of 10 MOS type chemical sensors to JW fermented by different pretreatment methods are significantly (*p* < .05) classifiable, because most plots of each aroma indexes in the diagram are not overlapping each other, illustrating different pretreatment methods have significant effects on the aroma of JW. Thereinto, among all different pretreatment methods, JW fermented WP exhibited significant higher (*p < *.05) response values of WC sensors for aromatic compounds (W1C, W3C, and W5C) and lower response values of WW sensors (W1W and W2W) for sulfur organic compounds as well as WS sensors (W1S, W2S, W3S, W5S, and W6S) of broad‐range sensitivity than JW fermented by other pretreatment methods. Sulfur organic compounds have a high volatility and low thresholds, which mainly contribute to unpleasant aromas in wines (Mestres, Busto, & Guasch, 2002). This result indicated that JW fermented WP could produce more aromatic compounds and effectively reduce the content of deficient aroma than other pretreatment methods, which leads to a significant improvement in the overall aroma quality of JW. It is worth mentioning that the aroma intensity of JW fermented by J was significant lower (*p* < .05), suggesting pulp and peel contact could strengthen the intensity and complexity of aroma, which have been attested in the researches of Li, Lim, Yu, Curran, and Liu (2013) and Zhang et al. (2016).

It can be observed in Figure 4b that the difference of JW fermented by different pretreatment methods in the taste index of sourness is large with a range of 2.28, while the difference in bitterness, astringency, saltiness, umami, aftertaste‐a, aftertaste‐b, and richness is small (range < 1). Hence, it can be seen that the taste difference of JW fermented by different pretreatment methods was mainly reflected in sourness. Sourness, as a basic taste, too high or too low will lead to sharp or imbalance taste, affecting the quality of drinks. The higher response value of sourness in JW fermented WOP and JW fermented WP may be due to the higher acid content in the peel and flesh of jujube.

In spite of the differences in other taste indexes, the differences will not be tasted by the human tongue, nor will they affect the consumers’ preferences, since their response values are <1 (Kobayashi et al., 2010). Interestingly, although they could not be tasted by humans, JW fermented WP was found to have a higher bitterness, astringency, aftertaste‐a, and aftertaste‐b by E‐tongue. Earlier literatures have suggested a strong positive correlation between phenols and astringency/bitterness in wine (Kallithraka, Kim, Tsakiris, Paraskevopoulos, & Soleas, 2011; Landon, Weller, Harbertson, & Ross, 2008; Vidal et al., 2003). This implies that JW fermented WP may have a higher TPC than the other 2 pretreatment methods.

### 3.4 Bioactive compounds and antioxidant activity analysis

The contents of bioactive compounds and antioxidant activity heavily depended on the composition of samples and conditions of the test methods, which cannot be adequately described with one single method due to many potential factors, two or more test methods based on different mechanisms are usually needed to simultaneously explain the bioactive compounds contents and antioxidant activity of samples. In the present research, a theoretical basis for preliminary evaluation of bioactive compounds contents and antioxidant activity in JW can be provided using TPC, TFC, TAC, DPPH radical scavenging activity, and ABTS cation radical scavenging activity, respectively.

**Figure 5:**
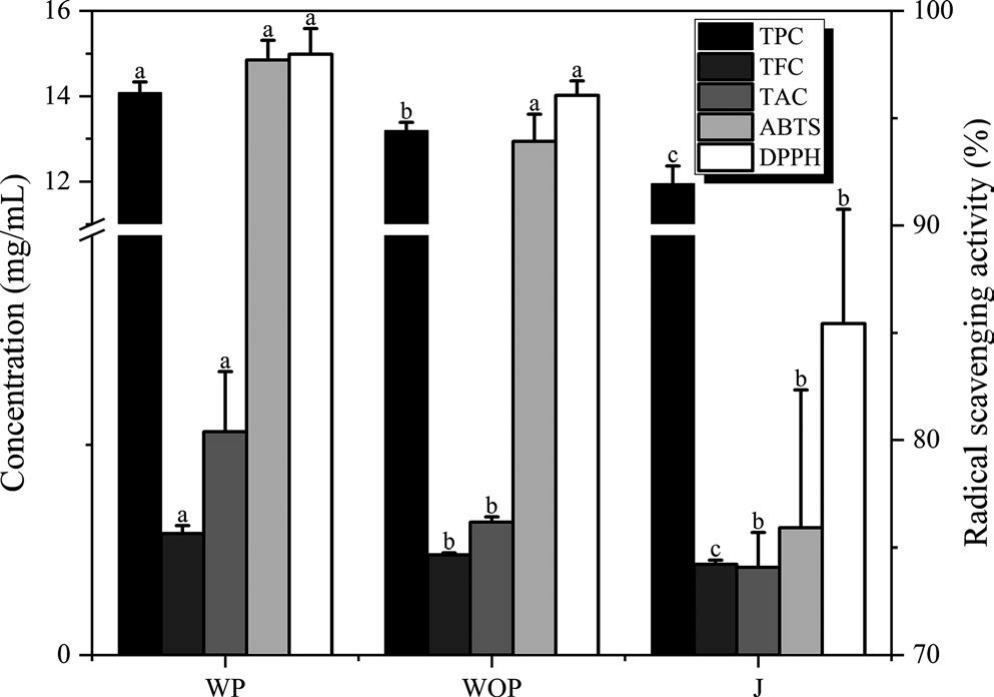
Total phenolic contents, total flavonoid contents, total anthocyanin contents, DPPH radical scavenging activity and ABTS cation radical scavenging activity of JW fermented by different pretreatment methods. Different letters indicate significant differences at *p* < .05

Results showed that JW has antioxidant activity and significant (*p < *.05) differences are observed in the levels of antioxidant activity in JW fermented by different pretreatment methods (Figure 5). It was generally found that DPPH and ABTS radical scavenging activity was closely related to the content of TPC, TFC, and TAC of the samples (Kwaw et al., 2018). In this research, the JW fermented WP contained significantly (*p < *.05) higher TPC, TFC, and TAC as well as exhibited significantly (*p < *.05) stronger DPPH and ABTS radical scavenging activity than the other two treatment methods, which also validates the conjecture about the antioxidant potential of JW fermented WP in the previous color and E‐tongue analysis. On the other hand, the weakest DPPH and ABTS radical scavenging activity was noted with the JW fermented by J having the lowest TPC, TFC, and TAC. This might result from the high contents of bioactive compounds and strong antioxidant activity in the peel and flesh (especially peel) of jujube (Xue et al., 2009; Zhang et al., 2010). Compared with other 2 pretreatment methods, the JW fermented WP can best preserve the bioactive compounds of jujube and has the strongest antioxidant activity.

It should be pointed out that although Gao et al. (2011) believed that the cultivar is the main factor determining the bioactive compounds and antioxidant activity in jujube. However, after comparing the bioactive compounds and antioxidant activity in different tissues of different jujube cultivars, Xue et al. (2009); Zhang et al. (2010) proved that the peel and flesh (especially peel) of all jujube cultivars had higher contents of bioactive compounds and stronger antioxidant activity. This suggests that the results obtained from the above might be of general significance and applicable to JW fermented by other cultivars of jujube.

### 3.5 PCA and CA

**Figure 6:**
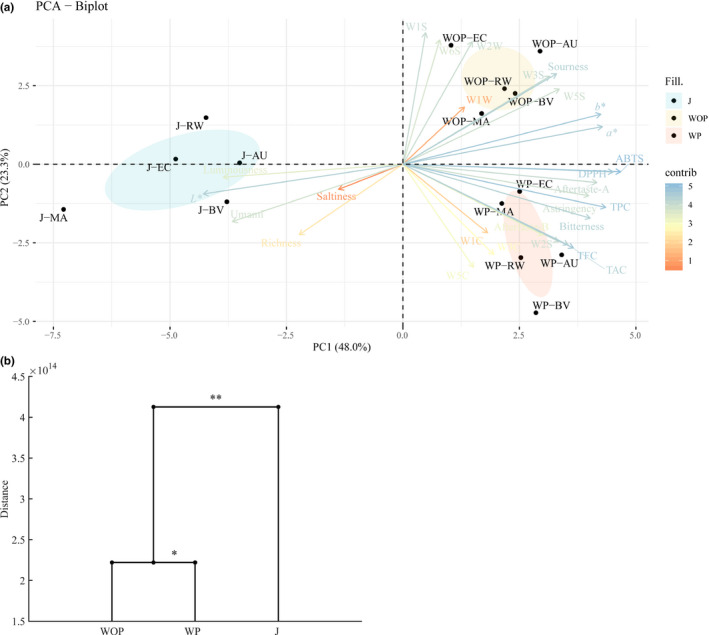
PCA biplot of JW fermented by different pretreatment methods (a). Dendrogram of JW fermented with different pretreatment methods calculated using Mahalanobis distances as well as MANOVA analysis. * and ** indicate significant differences at *p* < .05 and *p* < .01 level, respectively (b)

According to the 27 quality indexes, PCA, an unconstrained analysis using correlation matrix with ellipse confidence was performed to reveal how different pretreatment methods impacted the JW quality, and samples were depicted in the two‐dimensional plane as a PCA biplot (Figure 6a) (Rocha et al., 2020; Tang et al., 2020). The total variance (71.3%) was explained by the first two principal components, PC1 and PC2, accounted for 48.0% and 23.3% of the variance, respectively, led to a total variance of 71.3% altogether. As shown in Figure 5a, all the JW samples could be well distinguished into 3 clusters with 3 different pretreatment method, which proved JW quality is more affected by pretreatment methods, in consonance with CA based on UPGMA, PCoA, and CCA. The JW fermented by J are approximately located along the negative half of the x‐axis, and they are characterized by L*, luminousness, umami, richness, and saltiness. Both JW fermented WOP and JW fermented WP are situated on the right quadrant, in which JW fermented WOP are portrayed by their a*, b*, sourness, and WW sensors (W1W and W2W) for sulfur organic compounds and WS sensors (W1S, W3S, W5S, and W6S) of broad‐range sensitivity except W2S, while JW fermented WP are categorized by bitterness, astringency, aftertaste‐a, aftertaste‐b, TAC, TFC, TPC, DPPH, ABTS, and W2S sensor as well as WC sensors for aromatic compounds (W1C, W3C, and W5C). The same result could also be observed from abovementioned conventional analysis, E‐nose analysis, and E‐tongue analysis.

It is noteworthy that among the 3 clusters, the two‐dimensional distance between the cluster of JW fermented WP and the cluster of JW fermented WOP is minimum, demonstrates that the JW fermented with these two pretreatment methods share a more resemble overall quality.

Furthermore, data obtained above were evaluated by CA, a constrained classification of feature vectors into clusters via Mahalanobis distances as well as MANOVA analysis, to simultaneously compare the 3 independent variables (3 pretreatment methods) for each dependent variables (overall quality) (Granato et al., 2018). The dendrogram generated from CA providing a clear visualization of the relationships among the different pretreatment methods is shown in Figure 6b. Significant differences are observed in the overall quality of JW fermented by different pretreatment methods (*p* < .05). At the mean distance 2.2*10^14^ (*p* = .002 < .01), JW fermented WP and JW fermented WOP cluster; then at the mean distance 4.1*10^14^ (*p* = .015 < .05), JW fermented WP, JW fermented WOP and JW fermented by J cluster to become a group. Besides, a relatively little quality diversity between JW fermented WP and JW fermented WOP was noted, implies that the overall quality of JW fermented WP is more similar to JW fermented WOP rather than to JW fermented by J. This may be due to the relatively lower concentration of fermentation broth in juice, compared with pulp and peel. The results of CA are the same as the aforementioned CA based on UPGMA, PCoA, and PCA results.

### 3.6 Identification of indicators causing quality variations

**Figure 7:**
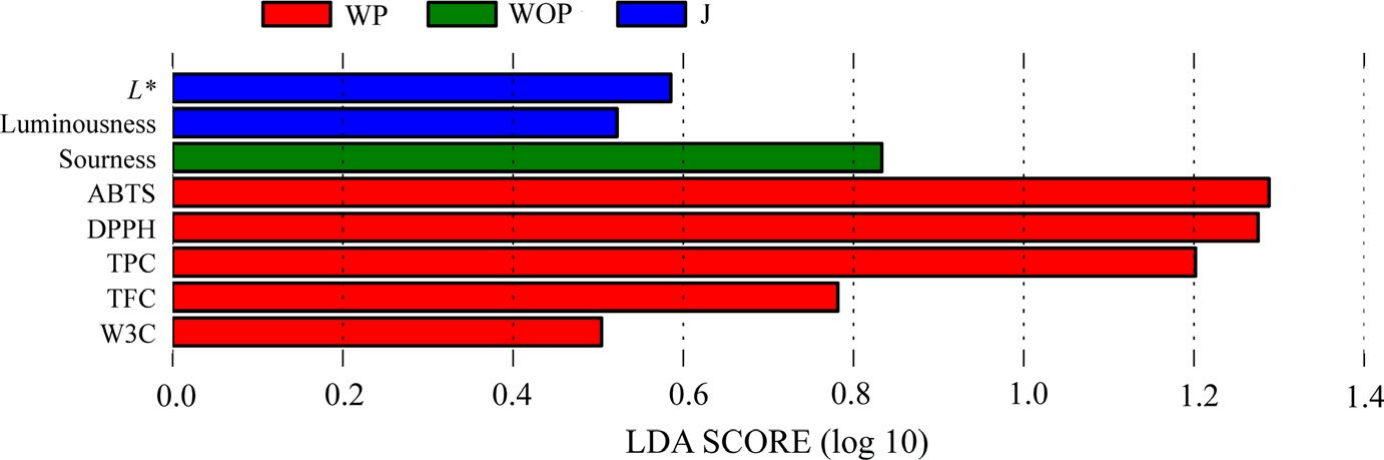
LDA score plot of the differentially abundant quality indexes among the JW fermented by different pretreatment methods. The threshold of the logarithmic LDA score was 0.4

To determine the specific quality indexes, which potentially able to explicate the dissimilarities among the JW fermented with different pretreatment methods (Segata et al., 2011). The LDA with a score threshold of 0.4 for discriminative features was applied (Figure 7).

Eight quality indexes including three color attributes and five aromas, namely L*, luminousness, sourness, ABTS, DPPH, TPC, TFC, and W3C were verified as indicators which cause quality variations among the JW fermented by different pretreatment methods. In JW fermented WP, bioactive compounds and antioxidant activity indexes (ABTS, DPPH, TPC, and TFC) have the highest LDA scores of 0.78 and higher, followed by sensor W3C of aromatic compounds with an LDA score of 0.5. Then, in JW fermented WOP, sourness was proved to be the dominant quality index with an LDA score of 0.83. Moreover, L* and luminousness were turned out to be the dominant quality indexes in JW fermented by J. This result indicates that different pretreatment methods brought about quality variations on the overall quality of JW, accordingly. As for other indexes that did not present in Figure 7, it can be concluded that they were not the dominant indicators and might not contribute greatly to the overall quality of JW fermented with different pretreatment methods.

### 3.7 Conclusion

In this research, the overall quality of JW fermented by 3 different pretreatment methods and 5 different starter cultures was evaluated; then, multivariate statistical analysis methods were used to assess the effects of pretreatment methods and starter cultures on JW. All in all, both pretreatment methods and starter cultures have effects on JW quality, in which pretreatment methods have much more significant effects.

The JW fermented by 3 different pretreatment methods were classified clearly by their overall quality, and that of JW fermented WP was the best among all. Compared with JW fermented WOP and JW fermented by J, JW fermented WP can not only enhance the color and flavor of the wine, but also maximizes the preservation of bioactive compounds and antioxidant activity of jujube.

## CONFLICT OF INTEREST

The authors have declared no conflict of interest.
